# Novel pharmacological and dietary approaches to target mTOR in B-cell acute lymphoblastic leukemia

**DOI:** 10.3389/fonc.2023.1162694

**Published:** 2023-04-14

**Authors:** Roberta Buono, Muneera Alhaddad, David A. Fruman

**Affiliations:** ^1^ Department of Molecular Biology & Biochemistry, University of California, Irvine, Irvine, CA, United States; ^2^ Hematology/Oncology Fellowship Program, CHOC Children's Hospital, Orange, CA, United States

**Keywords:** leukemia, B-ALL, tyrosine kinase inhibitors, mTOR, metabolism, nutrient restriction, fasting mimicking diet, targeted therapy

## Abstract

High-risk subtypes of B-cell acute lymphoblastic leukemia (B-ALL) are frequently associated with aberrant activation of tyrosine kinases (TKs). These include Ph+ B-ALL driven by BCR-ABL, and Ph-like B-ALL that carries other chromosomal rearrangements and/or gene mutations that activate TK signaling. Currently, the tyrosine kinase inhibitor (TKI) dasatinib is added to chemotherapy as standard of care in Ph+ B-ALL, and TKIs are being tested in clinical trials for Ph-like B-ALL. However, growth factors and nutrients in the leukemia microenvironment can support cell cycle and survival even in cells treated with TKIs targeting the driving oncogene. These stimuli converge on the kinase mTOR, whose elevated activity is associated with poor prognosis. In preclinical models of Ph+ and Ph-like B-ALL, mTOR inhibitors strongly enhance the anti-leukemic efficacy of TKIs. Despite this strong conceptual basis for targeting mTOR in B-ALL, the first two generations of mTOR inhibitors tested clinically (rapalogs and mTOR kinase inhibitors) have not demonstrated a clear therapeutic window. The aim of this review is to introduce new therapeutic strategies to the management of Ph-like B-ALL. We discuss novel approaches to targeting mTOR in B-ALL with potential to overcome the limitations of previous mTOR inhibitor classes. One approach is to apply third-generation bi-steric inhibitors that are selective for mTOR complex-1 (mTORC1) and show preclinical efficacy with intermittent dosing. A distinct, non-pharmacological approach is to use nutrient restriction to target signaling and metabolic dependencies in malignant B-ALL cells. These two new approaches could potentiate TKI efficacy in Ph-like leukemia and improve survival.

## 1 Introduction

B-cell acute lymphoblastic leukemia (B-ALL) is the most common childhood cancer (1). Overall, the 5-year survival rates for children with B-ALL have increased to 90% in the last decades. Combination chemotherapy remains standard of care, with dose adjustment and adjuvant targeted therapies added to regimens based on risk stratification. Despite the increase in B-ALL survival rates, relapse still occurs in 15 to 20% of patients with poor outcomes after relapse, making B-ALL a leading cause of cancer related death among children and adults (1, 2).

Philadelphia chromosome-positive (Ph+) B-ALL was the first high-risk (HR) subtype to be defined at genetic and mechanistic levels. Characterized by the t(9;22)(q34.1;q11.2) translocation (3, 4), Ph+ B-ALL cells express the aberrantly activated tyrosine kinase (TK) fusion protein BCR-ABL1 that drives signaling pathways promoting cell proliferation and survival (5). Adding tyrosine kinase inhibitors (TKIs) such as imatinib or dasatinib to a chemotherapy backbone has contributed to improved outcomes in Ph+ B-ALL (6–9).

With the advancement of cancer genomic profiling and sequencing studies, researchers have identified different mutations and genetic rearrangements that drive other subtypes of high-risk B-ALL (1). This includes subtypes with similar signaling and transcriptomic signatures as Ph+ B-ALL but lacking the Ph chromosome, and collectively termed “Ph-like” B-ALL.

## 2 Ph-like ALL

Ph-like ALL was initially identified among patients with poor clinical outcomes who were found to have alterations of the *IKZF1* gene (encoding the transcription factor IKAROS) that is also frequently deleted in Ph+ B-ALL (10). Based on additional similarities in tyrosine kinase-activated signaling pathways and gene expression profiles to those of patients with Ph+ B-ALL, this subtype was named Ph-like ALL (3, 10).

The genetic alterations found in Ph-like ALL have been divided into subgroups based on the kinase fusion or cytokine receptor involved. Whereas a 2016 report defined five subgroups (11), a more recent review from the Mullighan group (1) subdivided Ph-like B-ALL into four groups: (1) JAK family activating, including rearrangement of cytokine receptors (*CRLF2, EPOR, IL2RB*) and/or *JAK* mutations: (2) ABL-class including rearrangements of *ABL1, ABL2, PDGFRA, PDGFRB, or CSF1R*; (3) Other kinase, including *FLT3* and *NTRK3*; (4) RAS signaling, including mutations in *NRAS* or *KRAS*. The relative frequency of genetic alterations differs between pediatric and adult patients with Ph-like B-ALL. Adults have higher rates of *JAK2, EPOR*, and *IGH-CRLF2* rearrangements. On the other hand, pediatric as well as adolescent and young adult (AYA) populations have higher rates of other *CRLF2* rearrangements (such as *P2RY8-CRLF2*) and ABL-class gene rearrangements (12, 13).

The prevalence of Ph-like ALL differs by age, gender, race, ethnicity, and National Cancer Institute (*NCI*)-defined risk groups. A higher proportion of patients with Ph-like ALL are males with a male-to-female ratio of 2:1. With regard to ethnicity, Hispanic patients have a markedly higher prevalence of Ph-like ALL, with a particularly high association of *CRLF2* rearrangements (14). Ph-like B-ALL accounts for 10% of children with standard-risk (SR) B-lineage ALL and 15% of children of HR B-ALL (15, 16). The prevalence of Ph-like B-ALL increases with age, rising to 21% in adolescents and up to 27% in young adults with B-ALL, a rate almost 3 times higher than Ph+ B-ALL. In contrast to the rising incidence of Ph+ B-ALL with age, the incidence of Ph-like ALL seems to peak in the AYA population and decreases to 10% in older adults between the ages of 40 and 85 (11).

Patients with Ph-like ALL usually present with high leukocyte counts (>100,000/µL) at diagnosis and tend to have positive minimal residual disease (MRD) at the end of induction chemotherapy. Relapse rates are higher among patients with Ph-like in comparison with non-Ph-like B-ALL (17). In a completed phase 3 randomized controlled trial for HR-ALL (AALL0232) conducted by Children’s Oncology Group (COG), the 5-year event-free survival rate was lower among Ph-like B-ALL (63%) compared to non-Ph-like cases (86%) (11).

**Figure 1:**
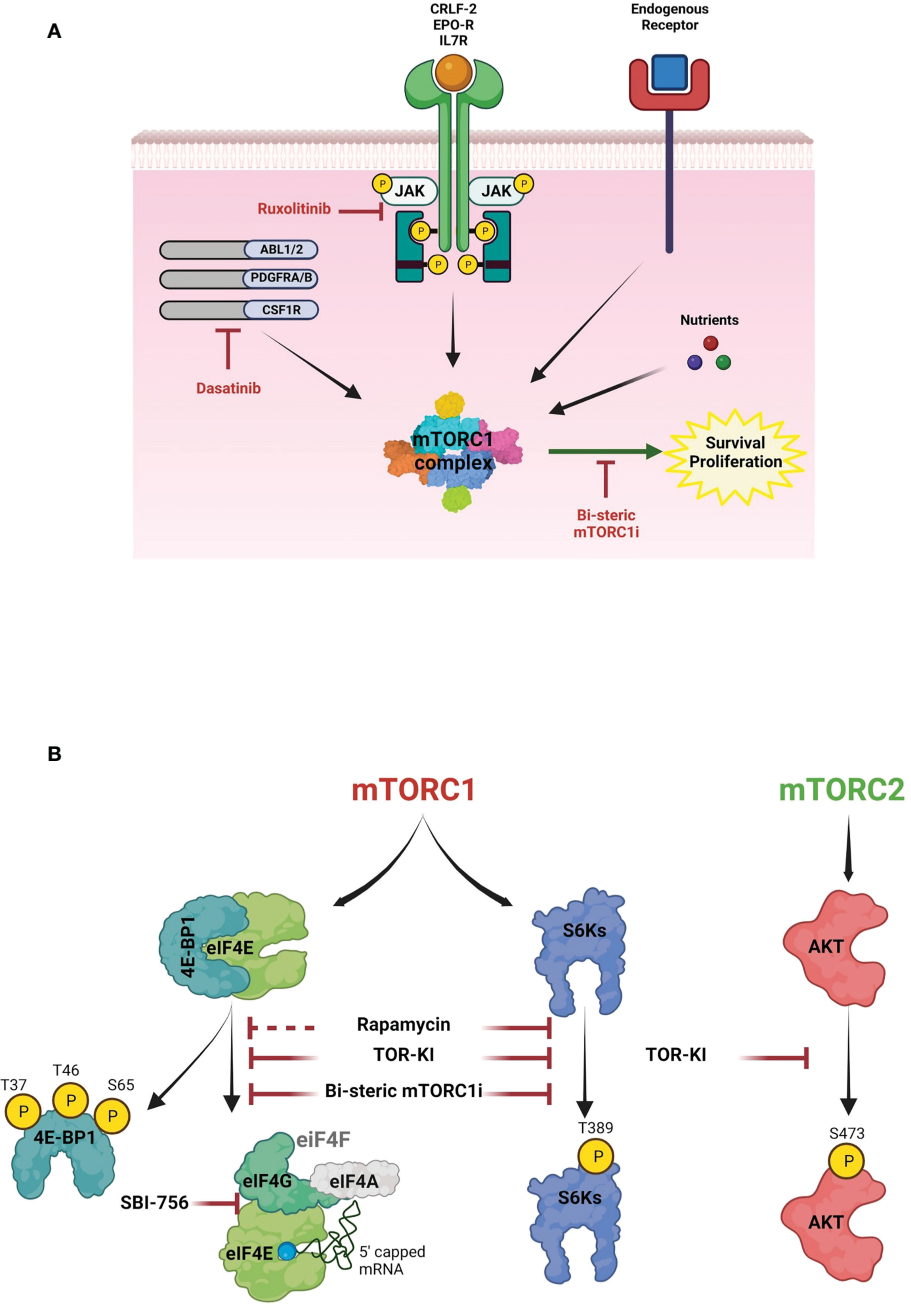
The role of mTOR signaling in B-ALL survival. **(A)** Schematic diagram of survival signaling pathways in B-ALL cells. mTORC1 contains the raptor subunit and other components that integrate signals from cytokine receptors, growth factors and nutrients to activate mTOR kinase activity. In Ph+ B-ALL, the BCR-ABL fusion tyrosine kinase (TK) increases activity of mTORC1 and other kinases. In Ph-like B-ALL, other genomic rearrangements (*CRLF2, EPOR, IL7R, ABL1, ABL2, PDGFRA, PDGFRB, CSF1R*) and/or JAK mutations activate TK signaling. Although clinically used TKIs (dasatinib, ruxolitinib) suppress oncogenic TK signaling, mTORC1 activity is largely maintained *via* nutrients and endogenous cytokine and growth factor receptors, thus promoting cell cycle progression and survival. Targeting this signaling with a selective bi-steric mTORC1 inhibitor (mTORC1i) has potential to enhance TKI cytotoxicity. **(B)** mTOR pathway. Two mTOR complexes, mTORC1 and mTORC2, have different substrates and together coordinate a complex network of anabolic pathways in response to signals from growth factors and oncogenes. Key substrates include ribosomal S6 kinases (S6Ks) and eIF4E-binding proteins (4E-BPs). mTORC1 phosphorylates 4E-BPs to promote eIF4F assembly. eIF4E binds to the 7-methylguanylate (m7G) cap present in most mRNAs and recruits the eIF4F complex to initiate cap-dependent translation. mTORC2 phosphorylates AKT and regulates cell proliferation and survival. First (rapamycin), second (TOR-KI) and third generation inhibitors (bisteric mTORCi) have different selectivity to inhibit the mTOR pathway. Notably, rapamycin is an allosteric inhibitor that inhibits phosphorylation of some substrates (e.g., S6Ks) more than others – particularly 4E-BP1. TOR-KIs act through an ATP-competitive mechanism to fully inhibit mTORC1 substrate phosphorylation but also inhibit mTORC2, thus suppressing AKT activity. Bi-steric mTORC1 inhibitors combine the mTORC1-selectivity of rapamycin with the complete kinase inhibition of TOR-KIs. The figures were created with BioRender.com.

Prior to the use of TKI therapy, Ph+ B-ALL was associated with very poor survival. With the early addition of imatinib or dasatinib to intensive chemotherapy regimens in the treatment of Ph+ B-ALL, there has been a significant improvement in the survival with or without hematopoietic stem cell transplantation in first complete remission (6, 7). Given the presence of actionable TKs in many Ph-like B-ALL tumors (15, 17), and early anecdotal case studies (18, 19), researchers were prompted to launch clinical trials testing the utility of TKI in the treatment of Ph-like B-ALL. The choice of TKIs is based on the underlying genetic mutation. Thus, patients with Ph-like ALL cases are divided into two major classes for therapeutic targeting purposes: ABL class (including *ABL1, ABL2, CSF1R,* and *PDGFRB* rearrangements) and JAK pathway activating (including *CRLF2, JAK2*, or *EPOR* rearrangements, *SH2B3* deletions, and *IL7R* indels) (**Figure 1A**). As described below, multiple clinical trials are currently investigating whether addition of the SRC/ABL/PDGFR inhibitor dasatinib or the JAK2 inhibitor ruxolitinib to the backbone of chemotherapy can improve the outcomes of children and adults with Ph-like ALL (13).

### 2.1 Dasatinib trials

Preclinical studies showed promising anti-leukemia activity of dasatinib in ABL class Ph-like B-ALL cells *in vitro* and in patient derived xenograft (PDX) models (3, 17, 20). In addition, several case reports demonstrated the efficiency of adding TKIs with chemotherapy in the management of children and AYA with refractory B-ALL (4, 18, 19, 21–25). Based on these findings, multiple clinical trials proposed the use of dasatinib with chemotherapy in newly diagnosed pediatric and AYA patients with ABL-class Ph-like B-ALL: the phase 3 COG trial AALL1131 (NCT01406756) and the SJCRH Total XVII trial (NCT03117751). Additional trials are testing imatinib or ponatinib TKIs with chemotherapy (NCT03007147, NCT03911128, NCT0450161). Results from these clinical trials are pending (16).

### 2.2 Ruxolitinib trials

Several studies reported the efficacy of JAK inhibitors (e.g., ruxolitinib) both in *CRLF2*-rearranged (*CRLF2*-r) and other JAK activated Ph-like B-ALL cells and PDX models (20, 26, 27). A phase 1 clinical trial conducted by the COG (ADVL1011) was able to study the side effects and best dose of ruxolitinib in treating young patients with relapsed or refractory solid tumor, leukemia, or myeloproliferative disease (28). Based on the promising results, a phase 2 clinical trial was conducted to test ruxolitinib in pediatric patients (1-21 years of age) with relapsed/refractory solid tumors and hematologic malignances. A phase 2 COG AALL1521 trial (NCT02723994) and SJCRH Total XVII trial is assessing the safety and potential efficacy of ruxolitinib with chemotherapy in newly diagnosed children and AYA with *CRLF2*-r/JAK pathway–mutant Ph-like B-ALL (NCT03117751). Finally, a recently opened phase 1 trial at the University of Chicago and other institutions (NCT03571321) is studying ruxolitinib in combination with the pediatric-inspired CALBG 10403 chemotherapy regimen (NCT00558519) specifically in AYA patients (18-39 years of age) with newly diagnosed Ph-like B-ALL with a planned phase 2 expansion if safety is demonstrated. These clinical trials are still open and have not reported final results (13, 16).

While ongoing clinical trials indicate that adding TKIs to multi-agent chemotherapy is safe (29), the extent to which TKI addition alone will improve therapeutic outcomes is not yet clear. Indeed, a trial by MD Anderson Cancer Center (MDACC) testing the efficacy of adding dasatinib or ruxolitinib with chemotherapy in adolescents and adults with relapsed/refractory Ph-like B-ALL (NCT02420717) was terminated early due to low accrual and lack of responses. Additional combination agents might significantly increase the depth and duration of response to TKI/chemotherapy regimens. Researchers continue to study TK-independent survival pathways that could be targeted to enhance the response to TKIs.

## 3 Targeting the mTOR signaling network

mTOR is an evolutionarily conserved serine/threonine kinase that forms two cellular complexes with distinct functions (30). mTORC1, containing the raptor subunit, integrates both cell-intrinsic (oncogene) and extrinsic (growth factors, cytokines, nutrients) signals to promote biosynthetic processes that support cell growth and proliferation (**Figure 1A**). Key substrates include ribosomal S6 kinases (S6Ks) and eIF4E-binding proteins (4E-BPs) that control the efficiency of mRNA translation (**Figure 1B**). mTORC2, containing the rictor subunit, links growth factor receptors to key serine/threonine kinases including AKT (**Figure 1B**).

There is a strong mechanistic rationale for targeting mTOR in high-risk B-ALL. Many blood cancer cells exhibit high mTORC1 activity, and phosphorylation of the mTORC1 substrate 4E-BP1 correlates with poor prognosis in B-ALL (20, 31).

The first class of mTORC1 inhibitor consists of the natural product rapamycin and synthetic analogs (rapalogs). Although rapamycin is a highly potent and selective mTORC1 inhibitor, it has an allosteric mechanism that completely blocks phosphorylation of some substrates (e.g. S6Ks) but has less effect on others – especially the gatekeeper for cap-dependent translation, 4E-BP1 (32–34) (**Figure 1B**). Dephosphorylation of 4E-BP1 reactivates its ability to disrupt the eIF4F translation initiation complex and suppress cap-dependent translation of mRNAs with key roles in proliferation and survival (35–37). Clinical studies of rapalogs added to chemotherapy have not clearly improved outcome in B-ALL (38–41). Second generation ATP-competitive mTOR inhibitors that completely inhibit both mTORC1 and mTORC2 (mTOR kinase inhibitors; TOR-KI) are more effective at causing dephosphorylation of 4E-BP1 to restore inhibition of eIF4F assembly (**Figure 1B**). We and others demonstrated superior efficacy of TOR-KIs versus rapalogs in combination with TKIs in Ph+ B-ALL and Ph-like B-ALL (34, 42–48). Two investigational TOR-KIs advanced to oncology clinical trials (TAK-228 (sapanisertib), AZD2014 (vistusertib)). However, clinical activity of these TOR-KIs has again been disappointing (49–53), likely due to toxicities associated with mTORC2 inhibition.

A third generation of mTOR inhibitors, termed RapaLinks or bi-steric mTORC1 inhibitors, were described first by Shokat and colleagues (53, 54) (**Figure 1B**). These compounds consist of rapamycin connected by a chemical linker to an ATP-competitive TOR-KI moiety. The rapamycin piece mediates high-affinity selective binding to mTORC1, while the TOR-KI portion binds in the active site. Bi-steric inhibitors have several advantages including potency (low nM activity in cells), selectivity for mTORC1 (> 10-fold versus mTORC2), strong reactivation of 4E-BP1 (**Figure 1B**), and stable pathway inhibition that allows for intermittent dosing *in vivo* (53, 54). A biopharma company, Revolution Medicines, has reported novel bi-steric mTORC1 inhibitor molecules with ~30-fold selectivity for mTORC1 and excellent *in vivo* properties (55), one of which (RMC-5552) has entered oncology clinical trials (NCT04774952) (56). We demonstrated that another bi-steric mTORC1 inhibitor (RMC-4627) has anti-leukemia activity in Ph+ B-ALL (57). Notably, a single weekly dose of RMC-4627 significantly enhanced dasatinib efficacy and tolerability in a Ph+ B-ALL xenograft model (57). A recent study by Shokat and colleagues demonstrated that RapaLink molecules are brain-penetrant, and that it is possible to confine pharmacological activity to the central nervous system (CNS) by combination dosing with a brain-impermeant rapamycin analog known as RapaBlock (58). This finding has potential relevance for improving maintenance therapy in B-ALL, which usually includes CNS prophylaxis (59–61).

## 4 Starvation, FMD, and other nutritional approaches

Developing novel chemical agents targeting oncogenic dependencies has led to some notable successes in precision oncology but is expensive and often results in a narrow therapeutic window and/or survival benefit. A distinct, non-pharmacological approach is to use nutrient restriction to target signaling and metabolic dependencies in malignant cells. Dietary interventions including fasting and fasting mimicking diet (FMD) have been applied successfully to treat solid tumor cancers in preclinical and clinical studies (62–65).

**Figure 2:**
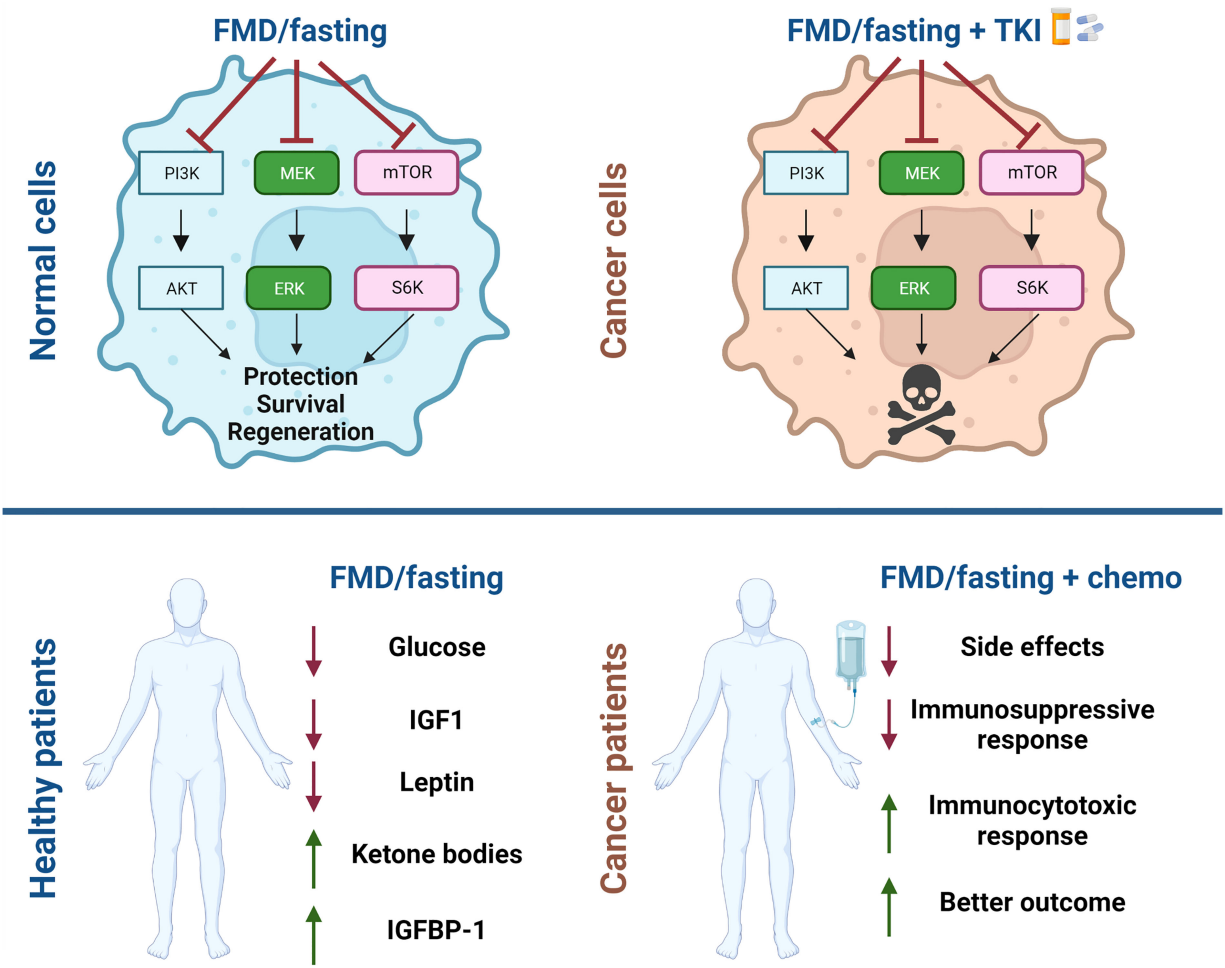
Schematic representation of beneficial effect of fasting and fasting mimicking diets for cancer treatment. Fasting and fasting mimicking diets (FMD) in combination with chemotherapy have a differential effect on normal cells and tumor cells. Normal cells enter in a protective mode while cancer cells are unable to survive. Systemic effects of fasting/FMD cycles are reduction in blood glucose, in circulating insulin growth factor-1 (IGF1) and leptin and increase in IGF1 binding protein-1 (IGFBP1) and serum ketone bodies. In combination with chemotherapy patients show fewer side effects, immunological changes and better outcomes. The figures were created with BioRender.com.

Mechanistically, nutrient restriction inhibits the intracellular nutrient sensor mTORC1, while activating AMPK to enforce catabolic reactions (such as glycogenolysis, proteolysis and lipolysis coupled to ketogenesis) with consequent activation of adaptive cellular stress responses (DNA repair, autophagy and antioxidant defense) (66). Nutrient restriction can suppress other signals such as PI3K/AKT and RAF/MEK/ERK (MAPK) pathways that cooperate with mTORC1 to promote oncogenesis (**Figure 2**). Together these cellular effects of nutrient restriction limit the ability of malignant cells to thrive and proliferate.

Fasting with water only, or chronic nutrient restriction, are dietary regimens that are difficult to sustain for long periods of time and cause nutrient deficiency with negative health consequences. For this reason, the Longo lab developed an intermittent FMD diet that can achieve the same anti-cancer benefits of fasting while providing some nutrients (67). FMD is a plant-based diet, that for each cycle of 4-7 days provides 34%–54% of normal caloric intake (11%–14% proteins, 42%–43% complex carbohydrates, 44%–46% fats) followed by FMD-free intervals (16–23 days) and has been applied successfully to treat different diseases like cancer, obesity and metabolic syndrome (68–75).

Patients undergoing FMD cycles manifest a reduction in blood glucose and in circulating insulin growth factor-1 (IGF1) and increase in IGF1 binding protein-1 (IGFBP1) and serum ketone bodies (66). In recent decades fasting and FMDs have been considered for the prevention and treatment of different solid tumors since their systemic effects have potential to enhance the efficacy of a wide variety of cancer treatments such as chemotherapy (**Figure 2**), immunotherapy and endocrine therapy (76). Importantly, these approaches have a differential effect on normal cells and cancer cells. Normal cells respond in ways that protect them against chemotherapy and other drugs, since in response to nutrient deprivation they arrest growth and activate stress resistance responses. This mechanism is referred to as differential stress response (DSR), while nutrient restriction weakens cancer cells by a process termed differential stress sensitization (DSS) (77). Cancer cells are sensitized by fasting/FMDs because of the constitutive activation of oncoproteins, which suppress stress resistance response and promote the generation of reactive oxygen species and induce cell death (63, 78). The effects of fasting/FMD in inducing DSS both in *in vitro* and *in vivo* models are mediated, in part, by reduction of circulating IGF-1 and glucose levels (**Figure 2**).

Fasting potentiates the anti-cancer activity of TKI in different types of solid tumors: breast cancer, colon rectal, and in non-small-cell lung cancer (79, 80). *In vitro*, short term starvation conditions increase the ability of commonly administered TKIs (erlotinib, gefitinib, lapatinib, crizotinib and regorafenib) to block cancer cell proliferation by inhibiting ERK phosphorylation and E2F-mediated transcription (79). Similar results were found by Lo Re at al., in a study showing that fasting potentiates the effect of sorafenib in a hepatocellular cancer mouse model by inhibition of MAPK signaling (80).

FMD cycles are also able to protect mice from PI3K/AKT and mTOR inhibitor-induced hyperglycemia, a common dose-limiting side effect of these inhibitor classes. In a mouse model of triple-negative breast cancer, a triple combination of FMD cycles with rapamycin and PI3K inhibitors (pictilisib, ipatasertib) resulted in long-term animal survival and reduction of side effects (71).

Among the beneficial effects of fasting and FMD is their ability to modulate immune response in the tumor site. These interventions boost tumor infiltration by CD8+ T cells and reduce immunosuppressive regulatory T cells (Treg) in syngeneic mouse models of melanoma and breast cancer. The immunomodulatory effects are mediated by downregulation of the cytoprotective enzyme heme-oxygenase HO-1 (70). A 48hr fasting period improves the efficacy of chemotherapy in a mouse model of mutant KRAS-induced lung cancers *via* activation of autophagy and reduction of Tregs in the tumor site (81).

Several clinical trials have evaluated the effect of fasting and FMDs in combination with chemotherapy as a therapeutic strategy in humans (73, 74, 82, 83). Patients diagnosed with different cancers that fasted voluntarily during their chemotherapy treatments reported fewer side effects and showed that fasting regimens were well tolerated. In the NCT03340935 trial that enrolled 101 patients with different type of neoplasms, five patients with advanced disease achieved complete and long-lasting tumor responses when treated with cycles of FMD plus the standard treatment (84). Another clinical trial (NCT03595540) evaluated the effect of FMD cycles on patients’ body composition and confirmed that FMD cycles are feasible and safe at low nutritional risk, with no negative impact on body composition and nutritional status in cancer patients. Some of the patients in this trial had hematological malignancies and three patients were also treated with TKI (74).

Similar to preclinical studies, FMD diet in human tumor patients reshapes anti-cancer immunity by downregulation of peripheral blood immunosuppressive myeloid cells and Tregs while increasing intratumor T-helper-1/cytotoxic responses, interferon-γ and other immune responses associated with better clinical outcomes. Together with good safety profile, the FMD had a global compliance rate of 91.8%, much higher of any other nutritional interventions (73). This high compliance rate is likely due to the short-term, intermittent regimen that includes adequate nutrition.

## 5 Diet and leukemia

While much of the work on dietary intervention in oncology has focused on solid tumors, there is increasing evidence for benefit in blood cancers. Fasting alone inhibits the initiation and reverses the leukemic progression of both B-cell and T-cell acute lymphoblastic leukemia (B-ALL and T-ALL, respectively), but not acute myeloid leukemia, in xenograft mouse models (85). Fasting suppresses leukemia progression in part through increased leptin receptor expression, which leads to the activation of the *PRDM1* gene encoding the BLIMP1 transcription factor. The expression of leptin signaling-related genes, at early stages, prevents the development of leukemia, and at later stages, is responsible for the terminal differentiation and depletion of B and T leukemic cells. This finding provided important initial evidence that fasting could be a treatment option for patients with leukemia (85, 86).

Weight and nutritional status have a large impact on cancer survival, and obesity is a risk factor for poor outcome not only for ALL but also for other cancers (87). Notably, obese children at the time of ALL diagnosis are more likely to relapse (88, 89). Obese mice with syngeneic ALL have poorer response to chemotherapy than control mice (90). Tucci et al. demonstrated that the switching to a low-fat diet together with vincristine treatment improves ALL outcome in a syngeneic Ph+ B-ALL obese murine model (91).

The benefit of caloric restriction *via* diet and exercise in ALL patients was shown in a prospective, nonrandomized, controlled IDEAL (The Improving Diet and Exercise) trial. 40 patients between 10 and 21 years old during their chemotherapy treatment were subjected to 20% calorie reduction obtained by nutrient restriction together with exercise. This intervention significantly reduced MRD risk, increased circulating adiponectin and reduced insulin resistance and had the potential to augment chemotherapy efficacy in newly diagnosed with B-ALL (92). A second randomized phase 2 IDEAL trial NCT05082519 (IDEAL2) is recruiting patients and will evaluate the beneficial effect of the IDEAL intervention in reducing chemoresistance in B-ALL patients.

## 6 Conclusions and perspectives

The discovery of Ph-like B-ALL and its association with aberrant TK activity has led to optimism for achieving better outcomes by adding precision TKIs (dasatinib or ruxolitinib) to the backbone of chemotherapy. However, even if TKI regimens achieve regulatory approval to prevent or treat relapsed/refractory patients, we can anticipate that some patients will still fail to achieve deep and durable remissions. One biological rationale for this statement is that leukemia cells exist in a complex microenvironment that provides survival signals independent of the driving oncogene(s) (**Figure 1A**). This idea is supported by the finding that aggressive B-ALL cells rapidly die when cultured *ex vivo*, unless provided with stromal cells and cytokines (93). Thus, TK activity alone is not sufficient to drive leukemia proliferation and survival. Various lines of evidence support the hypothesis that mTORC1 activity mediates TK-independent survival signaling: (1) mTORC1 activity can be partially sustained in the presence of TKIs through signaling input from endogenous receptors for cytokines and adhesion molecules, and through direct activation by amino acids (94); (2) Elevated mTORC1 activity is associated with poor prognosis (31); (3) Three generations of mTORC1 inhibitors can all enhance responses to TKIs in preclinical models of Ph+ and Ph-like B-ALL (57, 95).

Exploiting mTORC1 dependency has proven to be a long-term challenge with the first two generations of inhibitors failing to achieve a clear therapeutic window in clinical trials. Third-generation bi-steric mTORC1 inhibitors represent a new weapon in this fight, with a clinical trial of RMC-5552 already underway in solid tumors (NCT04774952). Through potent and selective mTORC1 inhibition, bi-steric inhibitors overcome limitations of both rapamycin and TOR-KIs. Compounds of this class deserve to be evaluated in detail in preclinical models of Ph-like B-ALL, to determine whether such agents can enhance efficacy of dasatinib and/or ruxolitinib.

In parallel, we propose that dietary intervention should be evaluated as a non-pharmacological approach. In particular, FMD is a safe, well-tolerated regimen of intermittent caloric restriction that has shown impressive rates of patient compliance in clinical trials. FMD can not only suppress mTORC1 activity in tumor cells, but also PI3K/AKT and MAPK pathways through both cell-intrinsic and systemic effects. As with any dietary intervention, there may be challenges to clinical implementation of FMD – especially in children and adolescents. However, integrative hospital care with nutritionists should enable compliance to the specified diet, especially in the in-patient setting.

The history of B-ALL treatment emphasizes the benefits of therapeutic combinations. Following the original success of combination chemotherapy to achieve cures in many patients, subsequent incorporation of tyrosine kinase inhibitors, immunotherapies and stem cell transplantation have further improved prospects for patients with high-risk disease. In this spirit, it makes sense to evaluate bi-steric mTORC1 inhibitors and nutritional interventions to maximize the potential benefit of TKIs in Ph-like B-ALL.

## Author contributions

RB and DF contributed to conceiving and designing the review. RB, MA and DF did the document retrieval. RB, MA, DF wrote the paper. All authors contributed to the article and approved the submitted version.
